# The effect of spatial structures on analogical problem solving

**DOI:** 10.3758/s13421-025-01817-7

**Published:** 2025-12-02

**Authors:** Amin Hashemi, Elisabet Tubau

**Affiliations:** Department of Cognition, Development and Educational Psychology, University of Barcelona, Pg. Vall d’Hebron 171, 08035, Barcelona, Spain

**Keywords:** Analogical problem solving, Spatial structures, Schematic drawing, Analogy creation

## Abstract

A critical step in analogical problem solving is recognizing structural similarities between the source (a known problem and its solution) and the target (the current problem). This task becomes particularly challenging when the source and target come from semantically distant domains. Previous research has suggested that global spatial configurations play an important role in detecting structural similarities. However, this effect has only been demonstrated in visual analogies. The present study aimed to investigate whether the salience of spatial structures is also relevant in verbal analogical problem solving. To this end, we manipulated the explicitness of the spatial relations described in the source narrative, as well as the method used for source processing (written summary, schematic drawing, or analogy creation). The results indicated that both the inclusion of explicit spatial features and schematic drawing enhanced analogical transfer. Schematic drawing was particularly effective when the narrative did not make the spatial properties explicit, suggesting that it promoted the inference of spatial relations. However, these effects only emerged when participants were informed about the relevance of the source. In contrast, analogy creation, which promotes the abstraction of causal relations leading to the solution, facilitated spontaneous analogical transfer. This effect was more pronounced when the source narrative included explicit spatial features. Therefore, while salient global spatial relations enhance the recognition of structural similarities between source and target analogs, understanding the causal relations underlying the solution supports successful analogical problem solving.

## Introduction

Analogy lies at the core of human cognition and is defined as a form of similarity in which common relationships are identified across different sets of elements (Gentner & Maravilla, 2018). Analogical reasoning involves two key steps: (i) recognizing a shared relational structure between two situations, and (ii) generating inferences based on these commonalities (Gentner, 1983; Gentner & Smith, 2013; Holyoak & Thagard, 1989; Hummel & Holyoak, 1997; Kokinov & French, 2003). Humans reason by analogy in both verbal and visual contexts. Visual analogies typically involve a set of images, one of which is missing. Solving such problems requires (i) perceiving the images, (ii) identifying relational patterns through image comparison, and (iii) mapping the inferred relations to determine the missing image (Chen et al., 2016; Lovett & Forbus, 2012; Sternberg, 1977; Stevenson & Hickendorff, 2018).

Recent studies have demonstrated that the global spatial structure of images, beyond the characteristics of individual objects, significantly influences visual analogical reasoning (Hashemi & Tubau, 2025; Lovett & Forbus, 2017; Matlen et al., 2020). Specifically, Hashemi and Tubau (2025) employed visual analogy problems that could be solved either by inferring global spatial rules (i.e., detecting changes in the overall shape of the images) or by identifying object-based rules (i.e., noticing changes in individual elements). Their results showed that participants predominantly relied on global spatial rules to solve the problems – especially when the global shapes were salient, such as objects arranged in circles or lines. Moreover, individuals with a stronger tendency to use global rather than object-based reasoning performed better on other analogical reasoning tasks. These findings suggest that sensitivity to the global structure of visual scenes may enhance the level of abstraction necessary for successful analogical problem solving. Given that similar analogical processes operate across both visual and verbal domains (Gentner & Smith, 2013; Kokinov & French, 2003; Markman & Gentner, 1993), global spatial structures may also play a critical role in verbal analogical problem solving.

Like visual analogical reasoning, verbal analogical problem solving involves several key steps: (i) constructing mental representations of the source (a textual description of a problem and its solution) and the target (a new problem solvable by analogy with the source); (ii) identifying shared relational structures between them; (iii) mapping correspondences between key elements; and (iv) adapting the known solution to the target problem (Chen, 1995; Gentner, 1989; Holyoak, 1984; Schunn & Dunbar, 1996). Thus, successful analogical problem solving depends on the construction of mental representations that support comparison and mapping between the source and the target.

Previous research has suggested that individuals may form visuospatial mental images of source and target narratives (Clement, 2009; Craig et al., 2019; Holyoak & Thagard, 1989; Reed, 2019; Schunn & Dunbar, 1996). In this way, the process of comparison and mapping in verbal analogies may resemble that of visual analogies. Consequently, as demonstrated in visual domains (Hashemi & Tubau, 2025; Lovett & Forbus, 2017; Matlen et al., 2020), the global spatial structure described in narratives may play a critical role in verbal analogical problem solving. However, this hypothesis has so far been supported only indirectly – through informal verbal reports (Gick & Holyoak, 1980) or via the use of visual schemas (e.g., Beveridge & Parkins, 1987; Gick & Holyoak, 1983; Pedone et al., 2001).

## Objectives and hypotheses

The main objective of this research was to directly examine the impact of global spatial structures on analogical problem solving by manipulating both the salience of the spatial relations described in the text and the mode of text comprehension. To this end, we employed the *Fortress Story* (Gick & Holyoak, 1980, 1983; Holyoak & Koh, 1987) as the source analog for solving the *Radiation Problem* (Duncker, 1945). As illustrated in the Appendix, the Fortress story conveys a clear spatial structure: a Fortress is located at the center of a country, with several roads radiating outward toward the periphery. Based on this scenario, we conducted three experiments in which we manipulated the explicitness of this spatial structure to test the following hypotheses.

First, Study 1 tested the hypothesis that a narrative with explicit spatial properties would enhance analogical problem solving compared to a narrative lacking such properties. As discussed above, spatial structures can play a crucial role in comparison and analogical transfer processes. Specifically, spatial descriptions that highlight broader spatial relations and global configurations may facilitate comprehension of the overall structure or “gist” of the scene (e.g., Oliva & Torralba, 2006). In contrast, narratives that lack explicit spatial features – or that emphasize rich local details – may lead individuals to focus on concrete elements and local spatial relationships, potentially hindering analogical mapping (Mani & Johnson-Laird, 1981).

Second, Study 2 examined the hypothesis that drawing a schematic representation of the source narrative would promote the explicit encoding of spatial relationships, thereby facilitating source–target comparisons and analogical transfer. Schematic drawings represent spatial layouts in a simplified, abstract manner (Lowe, 1999; Schnotz & Lowe, 2008). By reducing cognitive load and emphasizing structural relationships, such diagrams help extract essential spatial information while minimizing irrelevant details (Schnotz & Bannert, 2003). Encouraging individuals to conceptualize spatial scenarios at a higher level of abstraction has been shown to support more flexible and creative problem-solving strategies. Thus, it was expected that schematically drawing the narrated scenario would enhance analogical problem solving more effectively than writing about it. Moreover, this benefit was predicted to extend even to narratives with only implicit spatial relationships, by fostering the representation of a coherent spatial structure.

Finally, Study 3 explored the extent to which generating a new situation analogous to the Fortress story would promote spontaneous analogical transfer by fostering abstraction of the underlying causal structure that leads to the solution. While the effect of creating an analogous situation has been studied before, this activity has been introduced only after participants had encountered the target problem. Specifically, Minervino et al. (2017) found that participants who generated a problem analogous to the Radiation problem demonstrated greater success in analogical transfer from the Fortress story to the Radiation problem. Similarly, in the domain of mathematical problem solving, Bernardo (2001a, 2001b) showed that constructing a problem analogous to the source facilitated rule abstraction and spontaneous analogical transfer. Based on these findings, we hypothesized that, compared to writing a summary or drawing a diagram, the act of creating a structurally analogous problem to the Fortress story would promote a deeper understanding of its causal structure, thereby supporting spontaneous transfer. Furthermore, Study 3 also investigated whether the inclusion of explicit spatial features would enhance comprehending the underlying causal structure and further facilitate analogical transfer.

## Study 1

In this study, we developed an online task to examine the effect of explicitly including spatial information in the source narrative (the Fortress story) on analogical transfer. Specifically, we removed two sentences that conveyed spatial relations: “a fortress located in the middle of the territory” and “many roads radiated from the fortress to the towns around like the spokes of a wheel” (the full text of each version can be found in the Appendix). Gick and Holyoak (1980) identified the latter sentence as a critical element participants used to draw an analogy between the Fortress story and the Radiation problem. We hypothesized that the version without these explicit spatial cues would be less effective in facilitating solutions to the Radiation problem than the version that included them (i.e., the standard version).

### Method

#### Participants

One hundred and twenty-eight undergraduates participated in this experiment. The ethics committee of the University of Barcelona approved the research procedure. Participants were divided into two groups, each of which was assigned to one condition (see procedure).

#### Materials

As mentioned above, there were two versions of the Fortress story corresponding to the two conditions of this experiment. The first version was the Spanish translation of the story used first by Gick and Holyoak (1980; see the Appendix). The second version closely resembled the original Fortress story but the sentences referring to spatial properties: *“a fortress located in the middle of the territory”* and *“many roads radiated from the fortress to the towns around like e spokes of a wheel”* were eliminated and replaced by irrelevant information (see the Appendix). It is worth noting that the modified story remained sufficiently clear, not affecting the comprehension of the dispersion-convergence solution (see *Results* section).

In both conditions, we introduced a comprehension task related to the Fortress story. Specifically, participants were instructed to provide a brief summary of (i) the initial problem and (ii) the proposed solution described in the narrative.

#### Procedure

The task was administered via an online questionnaire created using Qualtrics. Participants were instructed to carefully read the Fortress story. They were then asked to write summaries of both the problem and the solution presented in the story, thereby completing a comprehension task. Next, participants engaged in an unrelated filler task lasting approximately 5 min.^1^ Following this, they were asked to solve the Radiation problem by submitting a written response. Finally, participants were prompted to reconsider the Radiation problem, this time with a hint encouraging them to reflect on the task they had initially completed.

#### Data analyses

The solutions to the Radiation problem were assessed based on whether they included the critical concept of weaker rays converging at the tumor. The scores assigned by the two raters exhibited a strong internal consistency, with an inter-rater reliability of.94. Furthermore, each participant’s summary of the story was scored. If their summary identified both the problem and its solution (i.e., the dispersion-convergence solution), they received a score of 1; otherwise, they received a score of 0. To investigate possible differences in outcome between different conditions, we used the Chi square test. Outcomes observed before and after the hint (indication about the relevance of the source) were compared separately. The datasets for all the studies are available via the Open Science Framework at the following link:

https://osf.io/63wsr/?view_only=a200f2793ac2445a82b41908fd5285c3.

### Results and discussion

Most participants in both groups demonstrated a correct understanding of the story, indicating that spatial properties did not significantly impact their comprehension of the story. The rate of correct comprehension was.92 in each condition. As expected, accuracy of the solution to the Radiation problem was lower for participants who did not show a complete understanding of the Fortress story (0% vs. 31%; χ^2^ (1) = 6.93, *p* =.008, φ =.18).

Table 1 presents frequencies and percentages of correct solutions to the Radiation problem across both experimental conditions. Before the hint, instances of spontaneous analogical transfer were rare. However, after the hint was provided, the success rate of analogical transfer was significantly higher in the condition featuring the Fortress story with rather than without explicit spatial cues (χ^2^(1) = 5.5, *p* =.02, φ =.20; see Table 1). This effect remained statistically significant even after excluding participants who demonstrated incomplete comprehension of the Fortress story (34% vs. 15% for the versions with and without explicit spatial cues, respectively; χ^2^(1) = 5.56, *p* =.02, φ =.22). This finding suggests that narratives that promote the representation of global spatial structures facilitate analogical transfer, supporting the hypothesis about the importance of global spatial representation in analogical problem solving. However, consistent with previous research (Gick & Holyoak, 1980, 1983), this benefit emerged only after the hint was given. Studies 2 and 3 further investigated alternative methods to enhance spontaneous analogical transfer.

**Table 1:** Frequency of correct solutions to the Radiation problem (% within parentheses) before and after the hint and the number of participants (N) for each Fortress condition in Study 1

Fortress spatial features	Before hint	After hint	Total	N
Explicit	3 (5%)	20 (31%)	23 (36%)	64
Not explicit	4 (6%)	9 (14%)	13 (20%)	64

## Study 2

Study 1 demonstrated that including explicit spatial properties in the source narrative influenced analogical transfer, supporting the idea that constructing a spatial mental representation of the scenario may play a pivotal role in the transfer process. Specifically, the ability to mentally represent the global spatial structure of the situation appears to facilitate the recognition of structural similarities between the source and the target, thereby promoting the analogical transfer of the solution. However, this effect was modest and only emerged after participants were given a hint.

This finding prompted us to explore alternative strategies for fostering analogical transfer beyond the use of explicit spatial cues. Prior research suggests that drawing can help individuals construct schematic spatial representations of a situation (Bobek & Tversky, 2016; Gagnier et al., 2017; Tversky, 2005). Building on this, we investigated whether prompting participants to draw, rather than write about, the source story (i.e., the Fortress story) would enhance abstraction and promote spontaneous analogical transfer. In Study 2, participants were asked to produce a schematic drawing of the Fortress story as part of the comprehension task, instead of providing a written summary.

Catrambone et al. (2006) previously found that drawing did not significantly outperform writing a summary in facilitating analogical transfer. However, participants who drew tended to exhibit a higher rate of spontaneous transfer than those who wrote. Given the relatively small sample sizes in their study – only 21 participants were included in the control condition – it is possible that clearer effects might emerge with larger samples. Furthermore, it is important to note that their study employed the standard version of the Fortress story, which already contained explicit spatial information. These spatial cues may have been sufficient for participants to construct a spatial mental representation of the narrative, thereby limiting the added benefit of drawing.

In Study 2, we used the same two versions of the Fortress story (with and without explicit spatial properties) as in Study 1. This time, however, participants were asked to produce a schematic drawing instead of writing a summary. We hypothesized that, for the version of the Fortress story lacking explicit spatial cues, drawing would help participants construct a spatial mental representation and thereby compensate for the absence of spatial detail. Consequently, we expected the rate of analogical transfer to be significantly higher among participants who created a visual schema compared to those who wrote a summary. For the standard version of the story (with explicit spatial cues), we expected to replicate the findings of Catrambone et al. (2006), that is, drawing and writing would result in comparable rates of analogical transfer after the hint. Nonetheless, we anticipated that drawing might still promote greater spontaneous transfer than writing.

Additionally, we analyzed the schematic drawings produced by participants. As noted by Mani and Johnson-Laird (1981), narratives that include unambiguous and explicit spatial properties provide clear information about both the spatial structure and the positions of elements. Therefore, we expected that drawings based on the version without explicit spatial properties would show greater variability in spatial representations compared to drawings based on the standard version.

### Method

#### Participants

One hundred and four undergraduates, who had not participated in the previous study, participated in this experiment. The ethics committee of the University of Barcelona approved the research procedure. Participants were evenly divided into four groups, each of which was assigned to one condition (see *Procedure*).

#### Materials

In Study 2, we utilized the same versions of the Fortress story (with and without explicit spatial properties) that were presented in Study 1 (please refer to the Appendix for details). After reading the story, participants were instructed to create schematic drawings representing both the problem and the solution of the Fortress story. Participants had the option to draw on paper or use computer programs to create their drawings. They were then required to upload their drawings, and for those who opted for drawing on paper, they had to take a photo of their drawing and upload it to the online platform.

#### Procedure

The task was also implemented as an online questionnaire via Qualtrics. Participants were asked to read the story carefully. Thereafter, participants draw a schema representing the problem and one representing the solution described in the Fortress story and uploaded the corresponding file. Then, they performed the same unrelated task used in Study 1, after which they were asked to solve the Radiation problem by writing their answers. Finally, using a hint, they were asked to rethink the Radiation problem by considering the task they had initially completed.

#### Data analysis

Solutions to the Radiation problem were evaluated according to the same criteria as in Study 1. To investigate possible differences in outcome between different conditions, we used the Chi square test.

### Results and discussion

Table 2 presents a comprehensive summary of the results from Study 2. Before the hint, participants who drew based on the Fortress story with explicit spatial properties tended to exhibit higher rates of spontaneous analogical transfer than those who drew based on the version without spatial cues, although this difference was not statistically significant. However, unlike the findings from Study 1, the rate of analogical transfer after the hint was comparable across both conditions. This result supports our hypothesis that drawing can compensate for the absence of explicit spatial information in the simplified version of the Fortress story. In other words, the act of drawing appeared to prompt participants to construct a spatial mental representation of the scenario, even when explicit spatial relations were not provided.

**Table 2:** Frequency of correct solutions to the Radiation problem (% within parentheses) before and after the hint and the number of participants (N) for each Fortress condition in Study 2

Fortress spatial features	Before hint	After hint	Total	N
Explicit	8 (16%)	17 (34%)	25 (50%)	50
Not explicit	4 (7%)	19 (35%)	23 (42%)	54

**Fig. 1:**
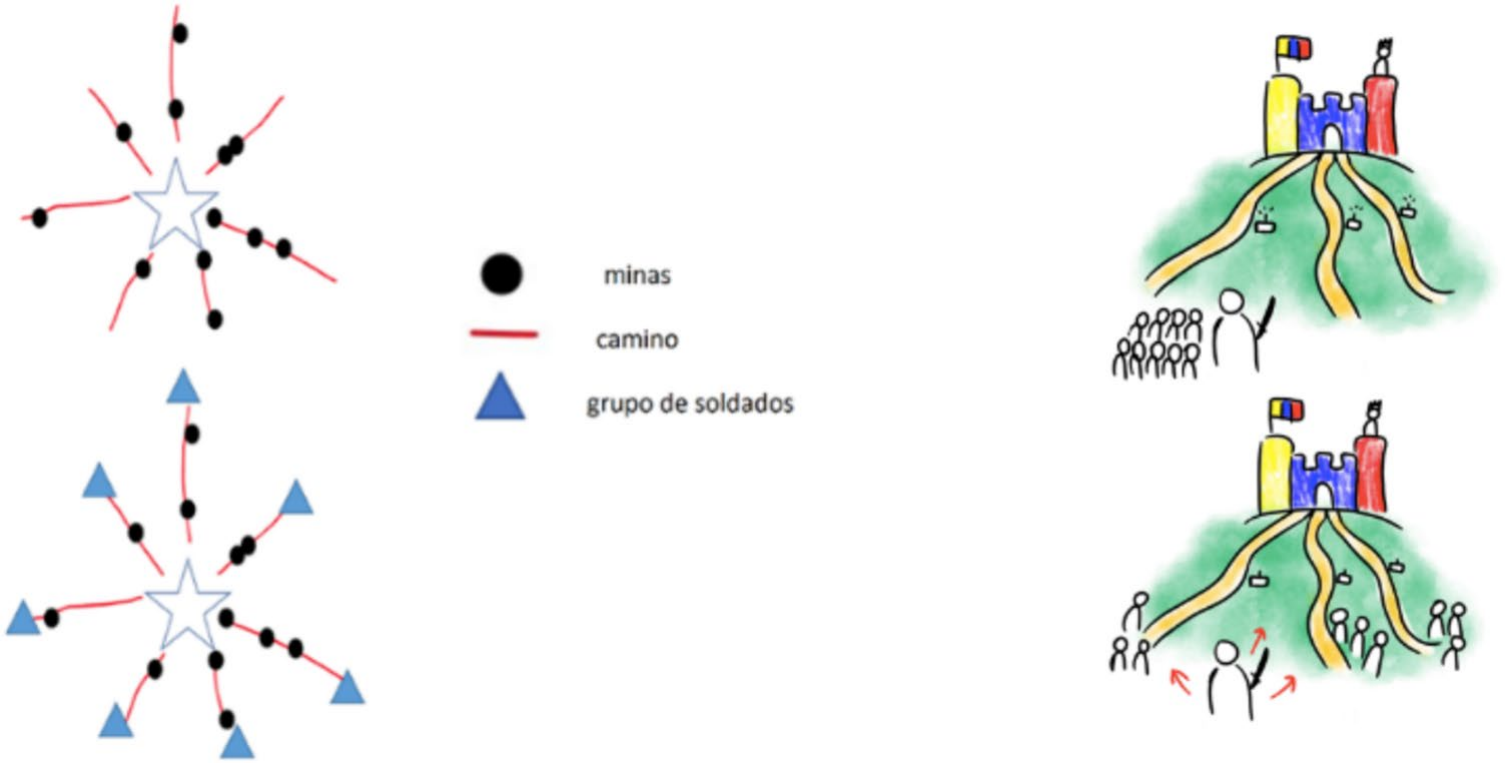
**Left:** A participant’s drawing of the problem and the solution of the Fortress story with spatial properties (legend: mines, path, group of soldiers). **Right:** A participant’s drawing for the Fortress story without spatial properties

As illustrated by the examples in Fig. 1, drawings from participants exposed to the version with explicit spatial cues typically reflected the described global spatial structure (left panel). In contrast, participants who drew the version without spatial cues still constructed spatially organized representations (right panel), despite the lack of explicit guidance. This suggests that drawing may encourage spatial representations and support analogical reasoning even in the absence of explicit spatial information.

Comparisons with Study 1 revealed that, before the hint, drawing based on the Fortress story with explicit spatial cues led to a higher rate of spontaneous analogical transfer than writing (16% vs. 5%; χ^2^(1) = 4.16, *p* =.04). However, following the hint, and consistent with the findings of Catrambone et al. (2006), drawing and writing resulted in comparable rates of analogical transfer (34% vs. 31%). In contrast, for the version of the Fortress story without explicit spatial properties, drawing and writing yielded similar transfer rates before the hint (7% vs. 6%). Yet, after the hint, drawing led to significantly greater analogical transfer than writing (36% vs. 14%; χ^2^(1) = 7.28, *p* =.007, φ =.25). These findings support the hypothesis that drawing can compensate for the absence of explicit spatial information by encouraging the inference of spatial relations. As a result, drawing appears to facilitate analogical transfer even when spatial properties are not explicitly stated in the text.

#### Analysis of drawings

Participants’ depictions of the Fortress story generally fell into two main categories based on spatial structure. Some participants illustrated the story using a circular spatial layout (see Fig. 1, left), with the Fortress placed at the center and roads radiating outward in a circular pattern. Others employed a non-circular spatial structure (see Fig. 1, right), positioning the Fortress on one side (top, right, or left) and arranging the roads accordingly. A few participants submitted other responses, such as depictions with no roads, only one road leading to the Fortress, or schematic summaries presented as text instead of drawings. Based on these patterns, we categorized the responses into three distinct groups: (1) schematic drawings with a circular spatial structure, (2) schematic drawings with a non-circular spatial structure, and (3) other responses (e.g., incomplete drawings or written summaries).

As expected, the results showed greater variation in the spatial implicit scenario (i.e., the Fortress story without explicit spatial information) compared to the explicit scenario (where spatial details were provided). In the explicit condition, 42 participants drew circular structures, and eight produced other responses. Notably, none of the drawings featured a non-circular structure, suggesting that the narrative strongly guided participants toward a circular layout. In contrast, in the implicit condition, 24 participants drew circular structures, 20 drew non-circular structures (placing the Fortress at the top, right, or left), and ten produced other responses. No significant difference was observed in analogical transfer rates between drawings with circular and non-circular structures. It is worth noting that in all the successful cases, for either structure, the convergence of paths was clearly represented.

## Study 3

Study 2 showed that drawing, compared to writing, had a modest effect on spontaneous analogical transfer when the narrative included explicit spatial features, and significantly enhanced analogical transfer after the hint when such features were absent. Nonetheless, the overall rate of spontaneous transfer remained low, regardless of the presence or absence of explicit spatial cues. This finding suggests that understanding the spatial structure of the scenario is useful for recognizing structural similarities but not sufficient for spontaneous analogical transfer.

Substantial improvements in spontaneous transfer have been reported in contexts that promote a more dynamic mental representation of the scenario described in the Fortress story – such as through animated videos (Kubricht et al., 2017) – or in situations where participants take a more active role, for instance, by physically manipulating objects to simulate the solution (Catrambone et al., 2006). As argued by Kubricht et al. (2017), the perception of movement may facilitate the understanding of the causal structure underlying the solution. Thus, a deeper comprehension of the causal relations in the source narrative may be required to elicit spontaneous transfer.

In the domain of mathematical problem solving, prompting participants to generate an analogous problem has been found to support rule induction and analogical transfer (e.g., Bernardo, 2001a), particularly when key principles are embedded within the text (Bernardo, 2001b). Similar effects have been reported for both figural and numerical analogies (Harpaz-Itay et al., 2006). These studies suggest that inventing an analogous problem promotes abstraction of the critical relational structure, thereby facilitating its transfer to novel problems.

Building on this, in Study 3, participants were asked to generate a problem analogous to the Fortress story, either with or without explicit spatial features. We hypothesized that this task would encourage the abstraction of the causal structure and thus lead to higher rates of spontaneous transfer compared to Studies 1 and 2. Moreover, if global spatial features aid in the comprehension of the causal structure, we expected this effect to be amplified in the condition that included explicit spatial cues.

### Method

#### Participants

Eighty undergraduates, who had not participated in the previous studies, participated in this experiment. The ethics committee of the University of Barcelona approved the research procedure. Participants were evenly divided into two groups, each of which was assigned to one condition (see *Procedure*).

#### Materials

In Study 3, we utilized the same versions of the Fortress story (with and without explicit spatial properties) that were presented in Study 1 (please refer to the Appendix for details). After reading the story, participants were instructed to create a new problem that could be solved in an analogous way to the problem described in the Fortress narrative.

#### Procedure

The task was also implemented as an online questionnaire via Qualtrics. Participants were asked to read the story carefully. Thereafter, participants created an analogous problem and solution to the one described in the Fortress story. Then, they performed the same unrelated task used in previous studies after which they were asked to solve the Radiation problem by writing down their answers. Finally, they were asked to rethink the Radiation problem after a hint given to participants to consider the task they had initially completed.

#### Data analysis

The accuracy of participants’ solutions to the Radiation problem was assessed using the same criteria as in previous studies.

The invented scenarios (those that participants created after reading the Fortress story) were coded as 0 when neither the problem nor the solution was analogous, as 1 when only the problem was analogous (i.e., the scenario included obstacles blocking the main path to a goal but no solution, or a non-analogous one), and as 2 when both the problem and the solution were analogous (i.e., the scenario also described a solution involving a dispersion–convergence strategy). In addition, a spatial structure variable was coded as 1 if an analogous spatial structure (a central element surrounded by multiple paths) was described, and as 0 otherwise.

To examine differences in analogical transfer across conditions, as well as potential relationships with the quality of the created scenarios, a logistic regression on spontaneous transfer rates – defined as providing the correct solution to the Radiation problem prior to the hint – was conducted. According to the residual deviance, the best-fitting model was the one that included the following predictors: Fortress condition (with the explicit condition as the reference category), Invented scenario (0–2), Spatial structure (0–1), and the interaction between Fortress condition and Invented scenario. The difference between this model and the one without the interaction was significant (residual deviance: 91 vs. 85, df: 76 vs. 75, AIC: 99 vs. 95; χ^2^(1) = 5.43, *p* =.020). The interaction between Fortress condition and Spatial structure was not included because some category combinations contained only four observations (see Table 4), leading to unstable estimates (Vittinghoff & McCulloch, 2007). Furthermore, the deviance of the model including this interaction did not significantly differ from that of the best-fitting model (residual deviance: 84, df: 74, AIC: 96; χ^2^(1) < 1, *p* =.321). Logistic regression analyses were performed in R (version 4.5.1; Team, R.C. (2000-2025) using the glm() function with a binomial (logit) link.

### Results and discussion

Table 3 presents the percentage of correct solutions to the Radiation problem across conditions, while Table 4 summarizes the quality of the created scenarios. As shown in Table 3, the percentage of participants demonstrating spontaneous analogical transfer (i.e., solving the Radiation problem before receiving a hint) was substantially higher than in Studies 1 and 2. Spontaneous transfer tended to be higher in the Fortress condition with explicit spatial features than in the condition without them (χ^2^(1) = 3.23, *p* =.072, φ =.20; see Table 3) and, overall, it varied across levels of quality of the invented scenario (r(78) =.43, *p* <.001). The Fortress conditions did not differ in the quality of the invented scenario, but they differed significantly in how participants described the spatial structure. Those in the condition with explicit spatial features were more likely to describe it than those in the condition without such features (χ^2^(1) = 4.15, *p* =.042, φ =.22; see Table 4).

**Table 3:** Frequency of correct solutions to the Radiation problem (% within parentheses) before and after the hint and the number of participants (N) for each Fortress condition in Study 3

Fortress spatial features	Before hint	After hint	Total	N
Explicit	25 (63%)	5 (13%)	30 (75%)	40
Not explicit	17 (43%)	7 (18%)	24 (60%)	40

**Table 4:** Frequency of participants (% within parentheses) who created a non-analogous scenario (0), analogous problem without an analogous solution (1), both analogous problem and solution (2), and made explicit a spatial structure in each Fortress condition in Study 3

Fortress spatial features	Invented scenario			Spatial structure	N
	0	1	2		
Explicit	11 (28%)	9 (23%)	20 (50%)	11 (28%)	40
Not explicit	9 (23%)	11 (28%)	20 (50%)	4 (10%)	40

A logistic regression analysis of spontaneous transfer rates (before the hint), with Fortress condition (Explicit vs. Implicit), Invented scenario (0–2), and Spatial structure (0–1) as predictors, revealed a significant main effect of Invented scenario and a significant Fortress condition × Invented scenario interaction. Correct responses increased across the levels of the Invented scenario, but this effect was substantially weaker under the implicit Fortress condition. The main effects of Fortress condition and Spatial structure were not significant (see Table 5). Interactions involving Spatial structure were not tested in the final model due to unreliable estimates (see *Data analysis* section). This limitation prevented determining whether the influence of Invented scenario also depended on Spatial structure. More specific analyses addressing this issue are reported below.

**Table 5:** Results of the logistic regression model on spontaneous transfer (before the hint)

	OR	SE	CI	z	p
(Intercept)	0.17	0.76	0.03–0.637	−2.35	0.019
Condition-imp.	2.21	0.99	0.33–17.7	0.80	0.423
Invented scen.	9.29	0.64	3.11–40.8	3.51	<0.001
Spatial structure	0.66	0.78	0.13–3.07	−0.52	0.600
Cond.i × Inv.sc.	0.19	0.75	0.04–0.76	−2.19	0.028

*OR* odds ratio, *SE* standard error, *CI* confidence interval

To further clarify the observed significant interaction, follow-up analyses were conducted separately for participants who generated an analogous solution (20 in each Fortress condition) and for those who did not (20 in each Fortress condition). For participants who created an analogous solution, spontaneous transfer was higher for the Fortress condition with explicit spatial features compared to the one without (90% vs. 45%; χ^2^(1) = 9.92, *p* =.002, φ =.48). In contrast, for participants who did not create an analogous solution, no significant difference was observed between the Fortress conditions (35% vs. 40% for the with vs. without explicit spatial features, respectively).

As mentioned earlier, the small number of participants who explicitly described the spatial layout did not allow for reliable statistical testing in the logistic analysis. However, it is noteworthy that among participants who correctly solved the Radiation problem, the description of the spatial structure was more likely in the explicit Fortress condition than in the implicit one (nine of 25 vs. one of 17; OR = 8.6 [1.02–79.55], Fisher’s exact = 0.031). This pattern suggests that the benefit of the Fortress condition with explicit spatial features stemmed, at least in part, from its greater tendency to encourage the representation of spatial structures in the invented scenarios (see also Table 4).

In sum, compared to Studies 1 and 2, inventing an analogous scenario increased spontaneous analogical transfer, independently of the Fortress condition. Nevertheless, providing explicit spatial features in the source further enhanced spontaneous transfer, particularly among participants who invented both an analogous problem and solution. Moreover, the description of an analogous spatial structure in the invented scenarios was more frequent in the Fortress condition with explicit spatial features, especially among participants who successfully solved the Radiation problem. Overall, these findings suggest that spatial representations may foster spontaneous transfer by facilitating the recognition of structural similarities between semantically distant scenarios.

## General discussion

This research investigated the role of explicit spatial properties in analogical transfer from a source narrative – the Fortress story, which described a problem and its solution – to an analogous target problem, the Radiation problem. The central hypothesis was that explicit spatial properties would enhance analogical transfer by promoting the representation of the global spatial structure of the source.

As expected, Study 1 found that the Fortress story with explicit spatial properties significantly improved analogical problem solving compared to the version without such properties – though this advantage emerged only after a hint was provided. Study 2 demonstrated that drawing a visual schema was more effective than writing a summary when the Fortress story lacked explicit spatial cues, suggesting that drawing may compensate for the absence of such cues by encouraging participants to build a spatial mental representation of the scenario. However, this benefit was again observed only after the hint. For the Fortress story with explicit spatial features, drawing had a modest impact on spontaneous transfer but did not significantly improve analogical transfer rates after the hint compared to writing. Finally, Study 3 revealed that creating an analogous problem and solution of the source narrative had a general positive effect on spontaneous transfer (i.e., before the hint). This effect was particularly strong in the Fortress condition that included explicit spatial descriptions. These findings and their implications are discussed in the following sections.

### The importance of global spatial properties

Gick and Holyoak (1980) initially proposed that recognizing shared spatial relationships between the Fortress story and the Radiation problem could facilitate analogical problem solving, emphasizing the potential role of spatial structure in analogical transfer and suggesting avenues for future research. However, much of the subsequent work investigating spatial relations relied on visual diagrams as the source representation for the Radiation problem (e.g., Beveridge & Perkins, 1987; Gick & Holyoak, 1983), rather than manipulating the linguistic description of spatial properties within the narrative itself.

In the present research, we explored whether explicit verbal references to spatial relations within the Fortress story would influence analogical transfer. To this end, we removed the sentences describing the global spatial configuration (e.g., “The Fortress was situated in the middle of the country” and “Many roads radiated outward from the Fortress like spokes on a wheel”) in one version of the story. Across studies, the results consistently underscored the critical role of such spatial descriptions in facilitating transfer to the Radiation problem.

Overall, the findings reinforce the idea that global spatial properties, when embedded in a narrative, serve as powerful cues for analogical mapping and problem solving (Beveridge & Perkins, 1987; Gick & Holyoak, 1980). Nevertheless, our results also indicate that grasping the spatial structure alone is not sufficient to guarantee spontaneous analogical transfer – a limitation that is further examined in the following sections.

### The effect of schematic drawing

The role of drawing in learning and problem solving has been widely studied, though findings have often been mixed. Some research supports the idea that generating drawings can be an effective strategy to enhance learning and facilitate problem solving (e.g., Butcher, 2006; Fiorella & Mayer, 2015; Hall et al., 1997; Leopold & Leutner, 2011; Schwamborn et al., 2010; Van Meter & Garner, 2005). However, other studies have shown that drawing is not necessarily more effective than writing (e.g., Catrambone et al., 2006; Leutner et al., 2009; Snowman & Cunningham, 1975). These discrepancies suggest that the effectiveness of drawing may depend on factors such as the type of problem, task demands, or characteristics of the learner – as also reported in the context of mathematical problem solving (Cooper et al., 2018; Hegarty & Kozhevnikov, 1999).

In Study 2, we aimed to clarify this issue by comparing the effects of drawing versus writing a summary of the source story under two conditions: one in which the Fortress narrative included explicit spatial cues, and one in which such cues were absent. The results offer a potential explanation for inconsistencies in prior findings. In line with Catrambone et al. (2006), we found that for the Fortress story with explicit spatial properties, drawing did not significantly enhance analogical transfer compared to writing – after the hint. However, for the version without spatial properties, drawing was significantly more effective than writing. This suggests that when spatial cues are not provided explicitly, drawing helps participants to mentally construct a spatial representation, thereby improving transfer. In contrast, when spatial structure is already embedded in the narrative, the added benefit of drawing diminishes.

We had also hypothesized that schematic drawing might promote spontaneous analogical transfer by encouraging a more abstract representation of the source problem compared to writing. However, comparisons between Studies 1 and 2 showed that only drawing based on the Fortress with explicit spatial features produced a modest improvement in spontaneous transfer. This indicates that simply creating a schematic drawing may not be sufficient. One potential factor that might contribute to successful transfer – beyond the spatial structure – is the representation of movement, which is inherent to both the Fortress and Radiation problems. Future research should explore whether drawing’s effectiveness is also influenced by other factors such as the nature of the problem or the skills of the reasoners.

### The effect of inventing an analogous situation

In contrast to the modest effect of spontaneous transfer observed in Study 2, Study 3 found that inventing a new scenario analogous to the source was highly effective. As previously mentioned, the effect of creating an analogous version of the source has only been explored in mathematical problem-solving contexts (Bernardo, 2001a, 2001b). In the present paradigm (the Fortress and Radiation scenarios), Minervino et al. (2017) also reported a benefit from analogy creation but only based on the target (the Radiation problem). Therefore, as far as we know, Study 3 is the first to investigate the effect of inventing an analogy for the source (the Fortress story).

In Study 3, both versions of the Fortress story, with and without explicit spatial features, were also compared. The invented scenarios in each Fortress condition included a similar proportion of analogous problems and solutions (see Table 4). In both conditions, successful analogical transfer was strongly linked to the creation of an analogous problem. Accordingly, generating an analogous problem appears to enhance the abstraction of the constraint (e.g., the impossibility of using a single path), making it easier to compare with the target problem (Bernardo, 2001a, 2001b). However, the benefit of creating an analogous solution was significant only for the version of the Fortress with explicit spatial features. Further analysis suggested this was, at least in part, because participants were more likely to describe the spatial structure of the invented scenario when the original Fortress story included clear spatial elements.

Thus, in both Fortress conditions, inventing an analogous problem seemed to foster abstraction of the problem’s structure, improving spontaneous comparison with the target. However, transferring the invented solution was more effective when the invented scenario also mirrored the spatial structure of the original. It is important to note that the modest impact of spontaneous transfer observed in the drawing task (Study 2) was also present for the Fortress with explicit spatial features. Overall, these findings suggest that when individuals can abstract both the spatial layout and the causal structure of a problem, they are much more likely to spontaneously transfer the solution to a semantically distant target.

### Limitations and further directions

There are several limitations to the studies reported here, which may guide future research. First, we only used one target (the Radiation problem) and one source (the Fortress story). As noted in earlier research (Gick & Holyoak, 1980, 1983), both the scenarios and their convergence–dispersion solutions are inherently spatial. Future studies should consider using a variety of narratives that differ in the degree and type of spatial features. One key research question could be how the number and complexity of spatial elements in a narrative influence analogical transfer.

For instance, the number of elements in a verbal scenario might affect whether people form spatial or object-based representations and mappings. Just as the number of elements in a visual scene influences the emphasis on global versus local features in perception (Martin, 1979), the number of elements in a verbal narrative may similarly affect whether mapping relies more on spatial structures or object characteristics. Specifically, when a scenario includes few elements, local (object-based) features may dominate, whereas scenarios with many elements may encourage global (spatial) representations. The Fortress story is a clear example of this spatial tendency, as it involves multiple roads leading to the Fortress and several groups coordinating to attack, all of which contribute to a spatially rich representation.

Furthermore, within any given scenario, some relationships between elements have spatial characteristics while others do not. For example, in the Fortress story, the relation between the general and the Fortress (i.e., to destroy it) is not inherently spatial, while the relation between the soldier groups and the Fortress (i.e., approaching from different paths) clearly involves spatial movement. We suggest that the extent to which analogical transfer relies on spatial mapping may depend on the number and relevance of such spatial relationships. Even if the explicit spatial descriptions were removed from the Fortress story, the narrative would still involve implicit spatial relations due to the directional movement of the soldiers.

Lastly, our experiments were limited to behavioral data, meaning we could not directly observe the mental representations participants formed while reading the source and target stories. We recommend that future research investigates how readers construct and use spatial mental representations during comprehension and reasoning. Techniques such as eye tracking or neurocognitive methods (e.g., neuroimaging) could provide deeper insights. Recent work has examined how humans build and rely on cognitive maps for various tasks, including problem solving and navigation (e.g., Bellmund et al., 2018; Bottini & Doeller, 2020). Understanding how a global spatial mental structure is constructed and applied during analogical problem solving – especially in narrative contexts – could be a promising area for future study.

## Conclusion

This research represents the first systematic investigation into the role of spatial structures in analogical problem solving within textually described scenarios. The results showed that explicitly including spatial properties in a narrative significantly enhanced analogical transfer. Additionally, we found that other methods of encouraging spatial representation – such as asking participants to schematically draw the source scenario – facilitated the comparison of problems with similar spatial structures and improved the analogical transfer process. The findings also revealed that, beyond spatial representations, abstracting the causal relations – through tasks like inventing an analogous scenario – is essential for spontaneous transfer to occur. Overall, the results highlight the importance of abstracting both the global spatial structure and the causal relations underlying the source solution in promoting spontaneous analogical transfer between semantical distant domains.

## Data Availability

Https://osf.io/63wsr/?view_only=a200f2793ac2445a82b41908fd5285c3

## Appendix

### The standard version of the Fortress story

A small country was controlled by a dictator. The dictator ruled the country from a Fortress located in the middle of the territory. Many roads radiated from the Fortress to the towns around like spokes of a wheel. A general, leading a large army, rebelled and vowed to capture the Fortress. However, the general was informed that the dictator had planted mines on each of the roads in such a way that the passage of the entire army through one of them would cause the destruction of the road, soldiers, and surroundings. The general also knew that the mines would not go off with small groups of soldiers. Therefore, he divided his army into small groups so that each marched on a different path in a synchronized manner. In this way, the entire army arrived at the Fortress at the same time and the general was able to capture the Fortress.

### The Fortress story without explicit spatial properties

A small country was controlled by a dictator. The dictator ruled the country from a Fortress painted in the colors of the country’s flag. There were many paths from the different towns to the Fortress. A general, leading a large army, rebelled and vowed to capture the Fortress. However, the general was informed that the dictator had planted mines on each of the roads in such a way that the passage of the entire army through one of them would cause the destruction of the road, soldiers, and surroundings. The general also knew that the mines would not go off with small groups of soldiers. Therefore, he divided his army into small groups so that each marched on a different path in a synchronized manner. In this way, the entire army arrived at the Fortress at the same time and the general was able to capture the Fortress.

### The standard version of the Radiation problem

Suppose you are a doctor faced with a patient who has a malignant tumor in his stomach. It is impossible to operate on the patient, but unless the tumor is destroyed the patient will die. There is a kind of ray that can be used to destroy the tumor. If the rays reach it all at once at a sufficiently high intensity, the tumor will be destroyed. Unfortunately, at this intensity the healthy tissue that the rays pass through on the way to the tumor will also be destroyed. At lower intensities the rays are harmless to healthy tissue, but they will not affect the tumor either. What type of procedure might be used to destroy the tumor with the rays, at the same time avoiding destroying the healthy tissue?

## Footnotes

1 In all the studies we used the Cognitive Reflection Test (CRT) developed by Thomson and Oppenheimer (2016).

## Declarations

### Open practices statement

Data for the experiments are available upon request, and none of the experiments was preregistered.
